# Effects of Suilysin on *Streptococcus suis*-Induced Platelet Aggregation

**DOI:** 10.3389/fcimb.2016.00128

**Published:** 2016-10-17

**Authors:** Shengwei Zhang, Junping Wang, Shaolong Chen, Jiye Yin, Zhiyuan Pan, Keke Liu, Lin Li, Yuling Zheng, Yuan Yuan, Yongqiang Jiang

**Affiliations:** ^1^State Key Laboratory of Pathogen and Biosecurity, Beijing Institute of Microbiology and EpidemiologyBeijing, China; ^2^Department of Clinical Laboratory, Dongfang Hospital, Beijing University of Chinese MedicineBeijing, China; ^3^Urumqi Ethnic Cadres' CollegeUrumqi, China; ^4^State Key Laboratory of Toxicology and Medical Countermeasures, Institute of Pharmacology and Toxicology, Academy of Military Medical SciencesBeijing, China

**Keywords:** *Streptococcus suis* (*S. suis*), suilysin (SLY), platelet aggregation, Ca^2+^ influx, streptococcal toxic shock syndrome (STSS)

## Abstract

Blood platelets play important roles during pathological thrombocytopenia in streptococcal toxic shock syndrome (STSS). *Streptococcus suis* (*S. suis*) an emerging human pathogen, can cause STSS similarly to *S. pyogenes*. However, *S. suis* interactions with platelets are poorly understood. Here, we found that suilysin (SLY), different from other bacterial cholesterol-dependent cytolysins (CDCs), was the sole stimulus that induced platelet aggregation. Furthermore, the inside-out activation of GPIIb/IIIa of platelets mediated SLY-induced platelet aggregation. This process was triggered by Ca^2+^ influx that depend on the pore forming on platelets by SLY. Additionally, although SLY induced α-granule release occurred via the MLCK-dependent pathway, PLC-β-IP3/DAG-MLCK and Rho-ROCK-MLCK signaling were not involved in SLY-induced platelet aggregation. Interestingly, the pore dependent Ca^2+^ influx was also found to participate in the induction of platelet aggregation with pneumolysin (PLY) and streptolysin O (SLO), two other CDCs. It is possible that the CDC-mediated platelet aggregation we observed in *S. suis* is a similar response mechanism to that used by a wide range of bacteria. These findings might lead to the discovery of potential therapeutic targets for *S. suis*-associated STSS.

## Introduction

Streptococcal toxic shock syndrome (STSS) is a severe, invasive streptococcal infection associated with the sudden onset of shock, acute respiratory distress syndrome, renal failure, bacteremia, and disseminated intravascular coagulation (DIC). The Gram-positive bacterium *Streptococcus suis* serotype 2 (*S. suis* 2) is an emerging human pathogen. As well as causing disease in pigs, *S. suis* 2 can also cause serious enzootic infections in humans, which are associated with septicemia, meningitis, and endocarditis (Tang et al., 2006; Wangkaew et al., 2006). In 2005, China reported over 200 human cases of STSS that had an unusual clinical disease presentation and a mortality rate of up to 20% (Sriskandan and Slater, 2006). Thrombocytopenia and multisystem dysfunction, such as liver failure, heart failure, and disseminated intravascular coagulation (DIC were found in more than half of the STSS patients. Moreover, purpura and gangrenous changes are found to be the typical skin manifestations in STSS patients (Tang et al., 2006; Yu et al., 2006).

Platelet–bacterium interactions are known to be involved in bacterial-associated diseases, such as infective endocarditis (IE), DIC, and purpura gangrenosa. *Lactococcus lactis* that expresses either ClfA or FnbpA was shown to be 100 times more infective than the wild-type *L. lactis* strain in an animal model of IE (Que et al., 2001). *Helicobacter pylori* infections cause platelet activation in patients (Davi et al., 2005), and P-selectin-dependent platelet aggregation might contribute to *H. pylori*-associated thrombocytopenia in patients (Yeh et al., 2010). Mouse infection models suggest that *S. pneumoniae*-induced thrombocytopenia and DIC are caused by this bacterium's neuraminidase, which removes sialic acid from platelet proteins and is involved in platelet clearance from the circulation (Grewal et al., 2008). However, the interactions between platelets and *S. suis* and the underlying molecular mechanism(s) involved in these interactions remain poorly understood.

Bacteria interact with platelets through a variety of mechanisms (Cox et al., 2011). Previous studies have shown that bacteria interact with platelets by direct binding to adhesion factors such as GPIIb/IIIa (Miajlovic et al., 2010), GPIbα (Plummer et al., 2005), and Toll-like receptor 4 (TLR4) (Stahl et al., 2006), or by indirect binding to these adhesion factors via plasma proteins such as fibrinogen (Walsh et al., 2008), IgG (Fitzgerald et al., 2006), von Willebrand factor (O'seaghdha et al., 2006), or complement 1q (Ford et al., 1996). In addition, bacteria secrete toxins that activate platelets and cause platelet aggregation (Lourbakos et al., 2001). However, our knowledge of the molecular events by which bacterial toxins control platelet–bacteria interactions is rudimentary.

Suilysin (SLY) is a 497 amino-acid protein belonging to the cholesterol-dependent cytolysin (CDC) family, which has more than 20 members, including pneumolysin (PLY) and streptolysin O (SLO), which are expressed by *S. pneumoniae* and *S. pyogenes*, respectively. Like other CDC family members produced by Gram-positive bacteria, a classical feature of these toxins is their ability to create transmembrane pores in cholesterol-containing membranes, thereby causing cell lysis (Giddings et al., 2004; Xu et al., 2010). In this study, different from other bacterial cholesterol-dependent cytolysins (CDCs), we found that SLY was the sole stimulus responsible for platelet activation and aggregation induced by *S. suis*. We also found that the SLY-induced pore dependent Ca^2+^ influx triggered “inside-out” signaling to activate GPIIb/IIIa, which mediated SLY-induced platelet aggregation. Our findings extend the similar observations related to PLY- and SLO-induced platelet aggregation, which is triggered by pore dependent Ca^2+^ influx.

## Experimental procedure

### Ethics statement

The healthy donors who supplied blood for this study provided written informed consent in accordance with the Declaration of Helsinki. Approval was obtained from the Institutional Medical Ethics Committee of the Academy of Military Medical Sciences. This research was approved by Ethics Committee on Animal Experimentation of the Chinese Association for the Accreditation of Laboratory Animals Care (CAALAC), and it included the relevant local animal welfare bodies in China. The permit number for all the animal work [SCXK-(JUN) 2013-018] was obtained from the Institutional Medical Ethics Committee of the Academy of Military Medical Sciences, China. All efforts were made to minimize suffering in the animals employed in this study.

### Reagents

Monoclonal mouse anti-human antibodies, including fluorescein isothiocyanate (FITC) conjugated anti-CD42b (clone HIP1), FITC conjugated anti-CD41a (clone HIP8), and the isotype control antibodies were from BD Bioscience (USA). FITC conjugated mouse anti-fibrinogen antibody was from Abcam (USA). U73122, ML-7, Y27632, and eptifibatide acetate, which are inhibitors for phospholipase C (PLC), myosin light chain kinase (MLCK), rho-associated, coiled-coil-containing protein kinase (ROCK), and CD41a, respectively, were from Sigma Aldrich (USA). Cholesterol and EGTA were also purchased from Sigma Aldrich. Adenosine diphosphate (ADP) was from MP Biomedicals (USA). Quest Fluo-8 calcium fluorescence probe was from AAT Bioquest®, Inc. (USA). Wright's stain was from Beijing CellChip Biotechnology Co., Ltd., China.

### Strains and culture supernatant

Chinese virulent *S. suis* strains (05ZYH33 and 4) were isolated originally from an STSS patient (Pian et al., 2012). The *sly* or *mrp* isogenic mutants of 05ZYH33 (Δsly and Δmrp) were constructed in our previous studies (He et al., 2014; Pian et al., 2015). The Δmrp mutant was used as an irrelevant control for Δsly in this study. The Canadian avirulent strain 1330, which does not express SLY, was donated by Prof. Marcelo Gottschalk (Université de Montréal, Montreal Quebec, Canada). The European SLY^+^-associated virulent strains (4005 and s735) were donated by Dr. Henk J. Wisselink. Group A streptococcus (GAS) was the M1 type, E477. The above-mentioned bacterial strains were stored in our laboratory. *S. suis* strains were cultured in Todd-Hewitt broth (THB, BD Biosciences) at 37°C for 4 h (OD_600_ = 0.4, exponential growth phase) or 6 h (OD_600_ = 1.0, stationary growth phase) and then harvested for the next experiments. Bacterial culture supernatants were obtained by centrifugation of the cultures at 8000 rpm for 5 min, after which they was filtered through a 0.22 μm bacterial filter. The filtered supernatants were stored in −80°C for future use. In the experiment where bacterial cells were used as the stimulus, *S. suis* strains and GAS were washed three times with phosphate-buffered saline (PBS) by centrifugation at 8000 rpm for 5 min and were then re-suspended in PBS at a density of 2 × 10^9^ CFU/ml for use. Strains are listed in Table S1.

### Preparation of recombinant suilysin (rSLY), pneumolysin (rPLY), streptolysin O (rSLO), and factor H-binding protein (rFhb)

To express recombinant proteins rPLY and rSLO in *E. coli*, the ORFs encoding PLY and SLO proteins (removed the signal sequence) were amplified by PCR and were cloned into the expression vector pTrcHis and transformed into *E. coli* strain BL21(DE3). The cloned gene sequences were confirmed by DNA sequencing. For protein expression, *E. coli* were cultured in 1 l of LB media containing 50 μg/ml of ampicillin at 37°C until the culture reached mid-log growth phase (OD_600_ = 0.6–0.8), after which 1 mM isopropyl β-D-1-thiogalactopyranoside (IPTG) was added to the culture to induce expression of the recombinant protein. Subsequently, the bacteria were cultured continuously at 16°C overnight. The bacterial culture was then collected by centrifugation (HITICH, Japan) at 8000 rpm for 10 min and the cell pellet was sonicated for 30 min on ice to release the recombinant protein. Recombinant proteins were purified using Ni^2+^ affinity chromatography columns (GE Healthcare). The recombinant proteins rSLY (Lv et al., 2011) and the irrelevant control protein rFhb (Pian et al., 2012) used in this study were purified as reported previously. The bacterial strains, primers, and plasmids used in this study are listed in Table S1.

### Human platelets

Whole blood from the healthy donors, who had not used anti-platelet drugs within the previous 15 days, was withdrawn at the 307 Hospital, and the blood from each person was collected in a tube containing sodium citrate (final concentration, 3.2%) (Shannon et al., 2007). A total of 6 ml of whole blood was centrifuged at 900 rpm for 10 min in a horizontal centrifuge (Sigma Aldrich) to prepare platelet-rich plasma (PRP, 3 × 10^8^/ml). The platelets in the PRP were marked by FITC conjugated anti-CD41a (clone HIP8) and the purity was determined by flow cytometry analysis. Whole blood was recentrifuged at 3000 rpm for 5 min to obtain platelet-poor plasma (PPP), which was used as a control for the aggregation assays.

### Platelet aggregation in PRP

Before the platelet aggregation detection assay was performed, the purity of the platelets in the PRP was analyzed by flow cytometry. Contaminating leukocytes were not seen in the PRP isolated, and the purity of the platelets in the PRP was >99% (Figure S1). Platelet aggregation was determined in a 4-channel platelet aggregometer SE-2000 (Succeeder, China) as described previously (Keane et al., 2010). Aggregation is represented by changes in light transmission, and the light transmission of PPP was used as the baseline. PRP (270 μl) was added to a glass cuvette and incubated at 37°C for 1 min, and then one or other of 30 μl of SLY (final concentration was 1μg/ml), *S. suis* cells (2 × 10^8^ CFU/ml), 05ZYH33 supernatant (Sup), Δsly Sup, ADP (20 μM, as positive control), or PBS/THB (negative control) was added to the cuvette. The rSLY (1 μg/ml) possesses equal hemolytic activity to the 05ZYH33 supernatant (10%) used in this study (**Figure 3A**). However, The final concentration of SLY in 05ZYH33 supernatant, as deduced from our previous study (He et al., 2014), was ≈0.15 μg/ml. This might be due to the rSLY used in this study was not added β-ME to keep its high hemolytic activity for avoiding the negative effects of β-ME on platelets. Hemolytic activity detecting assay was performed as reported previously (Hao et al., 2013). The cuvettes were incubated at 37°C for 10 min. To block platelet aggregation stimulation, PRP (270 μl) was pre-incubated with 2.7 μl of EGTA (3 mM), U73122 (20 μM), eptifibatide (10 μM), or anti-CD62P antibody (15 μl) at 37°C for 10 min, or with 2.7 μl ML-7 (100 μM), or Y27632 (100 μM) for 60 min before being exposed to the stimulus. To test the effect of cholesterol, SLY (990 μl) was pre-incubated with of 10 μl cholesterol (100 μg/ml) at 37°C for 10 min before addition to the platelets. Data are expressed as the mean ± SD. The experiment was repeated three times independently, and blood from a different donor was used in each experiment.

### Observation of platelet aggregation in whole blood by microscopy

Whole blood (100 μl) was incubated with 10 μl of SLY (1 μg/ml) or other stimuli at 37°C for 10 min after which 5 μl of the blood sample was used to prepare a blood smear on a glass slide. After the blood smear had dried completely, Wright's stain was added to cover the blood smear and an equal volume of PBS (pH 6.4–6.8) was added 1 min later. Wright's stain and PBS were gently mixed and the blood smear was incubated with the mixture for 5 min. The slide was then washed gently with distilled water and subjected to microscopic analysis using an oil-immersion objective (Olympus, Japan).

### Measurement of platelet lactic dehydrogenase (LDH) release

LDH release was measured to evaluate the cytotoxicity of recombinant SLY, PLY, and SLO to platelets. PRP was incubated with the either one of the recombinant proteins and the supernatant was collected by centrifugation at 3000 rpm for 5 min. LDH in the supernatant was measured by a CytoTox 96® Non-Radioactive Cytotoxicity Assay (Promega, USA). Cell lysis buffer and PBS were used as the positive and the negative controls, respectively. The relative cytotoxicity (%) = [(OD_490_ sample − OD_490_ PBS) ÷ (OD_490_ positive control − OD_490_ PBS)] × 100%. Data are expressed as the mean ± *SD* of three independent experiments, with each experiment using blood from a different donor.

### Flow cytometry analysis of platelet activation

The surface GPIIb/IIIa was detected by Flow cytometry with FITC conjugated anti-CD41a (CD41a often referring to GPIIb, clone HIP8), as per a previous description (Parimon et al., 2013). Briefly, 100 μl of heparinized human blood was incubated with 15 μl of FITC conjugated anti-human CD41a at 37°C for 10 min. Next, rSLY or a different stimulus was added and the blood was incubated for another 15 min. One mL of red cell lysis buffer containing formalin was then added to the blood to lyse the red cells and to fix the platelets at room temperature for 8 min. After complete lysis of the red blood cells, the blood was centrifuged and the cell pellet was washed with PBS. The cell pellet was re-suspended in 300 μl of PBS and analyzed on an Accuri C6 flow cytometer (BD Biosciences, USA). The surface CD41a was determined by measuring the mean fluorescence intensity (MFI) of 10,000 events for each sample. To test the effect of cholesterol, SLY was pre-incubated with cholesterol (100 μg/ml) for 10 min and the blood was incubated with the mixture. To test the effect of EGTA, blood was pre-incubated with EGTA (3 mM) for 10 min and then incubated with rSLY or other stimuli. Fg binding to platelets was also analyzed by flow cytometry. Briefly, 100 μl of whole blood was incubated with 1 μl of FITC-conjugated anti-fibrinogen polyclonal antibody at 37°C for 10 min. The blood sample was then analyzed by flow cytometry. Representative histograms of the MFI for CD41a and fibrinogen binding are displayed. Data are expressed as the mean MFI ± *SD* of three independent experiments, with each experiment using blood from a different donor.

### Measurement of dense granule (or ATP) secretion

ATP secretion was determined to evaluate platelet activation after stimulation (Arman et al., 2014). Briefly, 270 μl of PRP was incubated with 30 μl of rSLY or 05ZYH33 supernatant or other stimuli at 37°C for 10 min. The supernatant was obtained by centrifugation at 5000 rpm for 5 min. ATP in the supernatant was measured using a luciferin-luciferase kit (CellTiter-Glo ® 2.0 Assay, Promega, USA), according to the manufacturer's manual. Data are expressed as the mean ± *SD*. The experiment was repeated three times and blood from a different donor was used in each experiment.

### Calcium influx to platelets

A total of 200 μl of PRP was added to each well of a 96-well microplate. The plate was centrifuged at 2000 rpm for 10 min and then washed twice with PBS. Next, 100 μl of calcium probe fluo-8 (5 μM) was added to the wells and the plate was incubated at 37°C for 30 min. The plate was centrifuged at 2000 rpm for 10 min and then washed twice with 200 μl of PBS to remove any calcium probe remaining in the solution. The platelets were re-suspended in 180 μl of HBSS (containing 2 mM Ca^2+^) or D-HBSS (without Ca^2+^), and then either 20 μl rSLY (1 μg/ml), rSLY (0.1 μg/ml), rSLY^P353V^ (1 μg/ml), cholesterol (100 μg/ml), or other control reagents were added. Ca^2+^ influx to the platelets was measured in a Varioskan Flash Multiplate Reader (Thermo, USA).

### *In vivo* infection

05ZYH33 and mutant Δsly were cultured at 37°C in 5% CO_2_ for about 6 h to stationary phase (OD_600_ = 1.0, ~1 × 10^9^ CFU/ml) and then centrifuged at 8000 rpm for 5 min. PBS was used to wash the bacteria and the dose was adjusted to ~1 × 10^9^ CFU/ml. Female BALB/c mice (6–8 weeks old) were challenged with 100 μl (~1 × 10^8^ CFU) of 05ZYH33 or the mutant Δsly bacteria through the caudal vein. Pathological changes were observed by hematoxylin and eosin (H&E) pathological staining for 48 h post-inoculation.

### Statistical analysis

Unless otherwise specified, all data are expressed as the mean ± standard deviation. All the platelet aggregation test assays were performed in PRP/human blood from three individual donors in independent experiments. Differences between two groups were assessed using an unpaired two-tailed Student's *t*-test where Levene's-test did not show statistical significance (*P* > 0.05); otherwise, non-parametric tests were used. For all tests, a value of *P* < 0.05 was considered as the threshold for significance. All statistical analyses were carried out using SPSS 15.0 (SPSS Inc., Chicago, IL, USA).

## Results

### SLY is the sole stimulus responsible for platelet aggregation induced by *S. suis*

**Figure 1:**
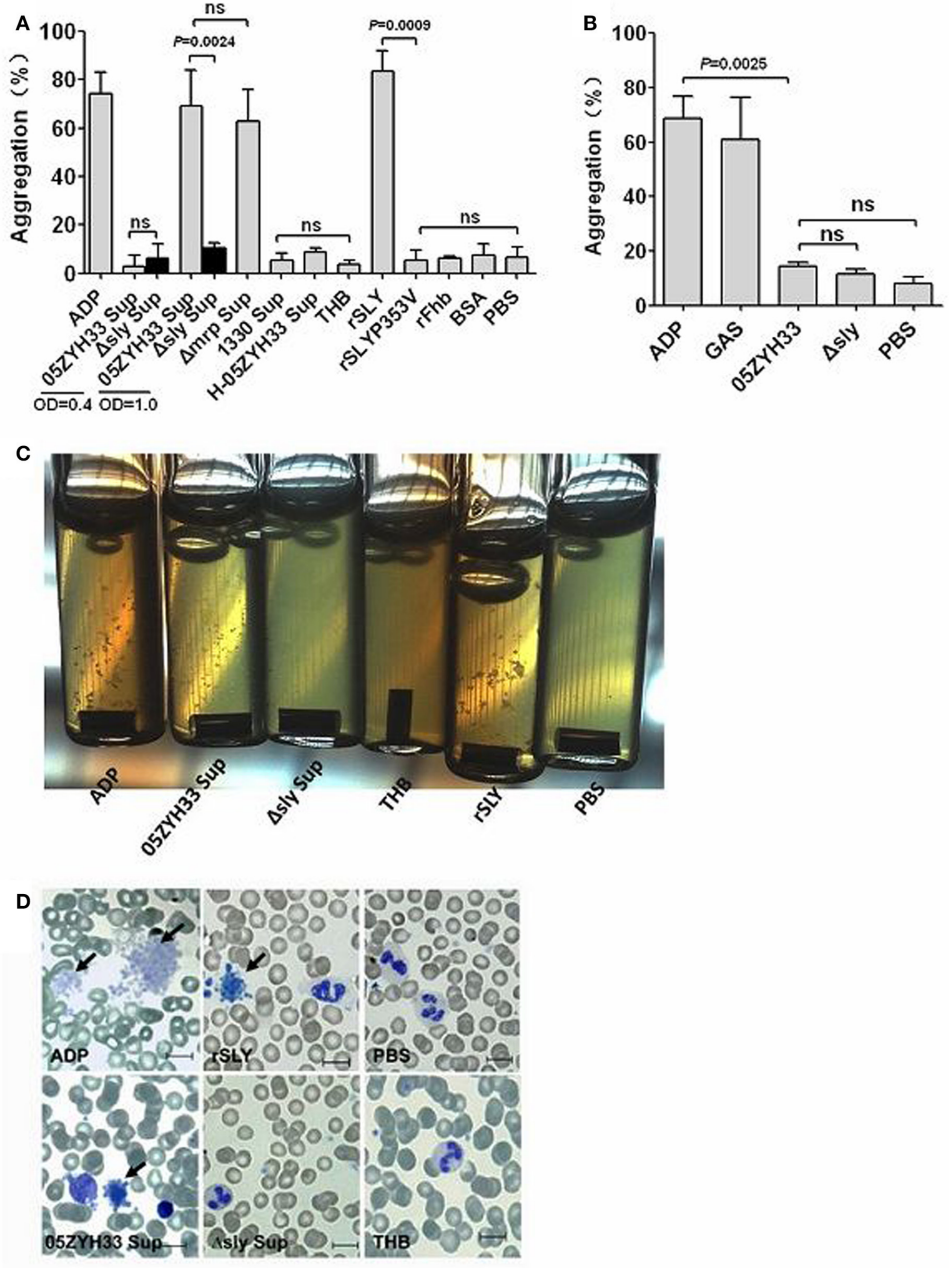
**SLY is the sole stimulus of ***S. suis***-induced platelet aggregation**. **(A)** The culture supernatant of *S. suis* and recombinant proteins or **(B)** the washed bacteria cells were added to PRP in a stirred cuvette. Platelet aggregation was expressed as a final percentage of light transmission detected by Platelet Aggregometer se-2000. ADP (20 μM) and GAS bacterial cells were used as the positive controls. Unpaired two-tailed Student's *t*-tests were used and the threshold for significance was *P* < 0.05; ns, not significant. Data are expressed as the mean ± *SD* of three independent experiments, with each experiment using blood from a different donor. **(C)** Platelet aggregations were observed when detecting the light transmission of PRP stimulated by bacterial supernatant (at OD_600_ = 1.0) or rSLY. **(D)** Wright's staining of cytospin preparations of *S. suis* culture supernatant or rSLY protein-treated whole blood samples. 05ZYH33, wild type strain; Δsly, isogenic mutant of sly; Δmrp, isogenic mutant of mrp (which was used as an irrelevant control for Δsly); 1330, SLY-negative Canadian strain; H-05ZYH33, heat-inactive 05ZYH33; Sup, supernatant; rSLY, recombinant SLY; rSLY^P353V^, recombinant non-hemolytic mutant of SLY^P353V^; rFhb, recombinant Factor H-binding protein, which was used as an irrelevant protein and was purified by the same procedure as that used for rSLY.

To elucidate the biological basis for stimulus-induced platelet aggregation, bacterial cells and stationary phase culture supernatants from *S. suis* strains were tested for platelet aggregation in human PRP. The 05ZYH33 stationary phase (OD_600_ = 1.0) supernatant significantly induced platelet aggregation, whereas the bacterial supernatant at exponential growth phase (OD_600_ = 0.4) and 05ZYH33 bacterial cells did not (Figures 1A,B), suggesting that there are secreted stimuli that induce platelet aggregation. SLY is an important secreted toxin of *S. suis* and high levels of it are produced at the end of the exponential growth phase (Gottschalk et al., 1995); therefore, we used it to test the aggregation-inducing activity of platelets. Interestingly, different from other bacteria, SLY seemed to be the sole stimulus responsible for platelet aggregation induced by *S. suis*. Because neither Δsly bacterial cells nor the supernatant were able to induce platelet aggregation (Figures 1A,B). Moreover, the supernatant from the SLY-negative Canadian strain 1330 culture and the heat-inactivated 05ZYH33 supernatant also failed to induce platelet aggregation (Figure 1A). Additionally, recombinant SLY (rSLY) stimulated platelet aggregation, whereas the non-hemolytic recombinant mutant SLY^P353V^ (rSLY^P353V^) and the irrelevant control protein rFhb did not (Figure 1A). Obvious platelet aggregation in the presence of the 05ZYH33 supernatant and rSLY was observed in aggregometer cuvettes (Figure 1C), and was also evident on blood smears with Wright's stain (Figure 1D). In SLY^+^ *S. suis*-associated Chinese and European strains, *S. suis* 1940 displayed poor hemolytic activity (He et al., 2014). Interestingly, besides *S. suis* 1940, the stationary phase (OD_600_ = 1.0) from other SLY^+^ *S. suis* strains significantly induced platelet aggregation, whereas the bacterial cells failed (Figure S2). Taken together, these results indicate that SLY is the sole protein involved in *S. suis*-induced platelet aggregation.

### SLY induced platelet aggregation occurred via GPIIb/IIIa

During platelet activation, the agonist induces a change in platelet shape, granular secretion, and inside–out signaling resulting in a conformational change in the extracellular domains of GPIIb/IIIa, thus allowing fibrinogen binding, while “outside-in” signaling is activated and regulates the extent of platelet aggregation (Zarbock et al., 2007).

**Figure 2:**
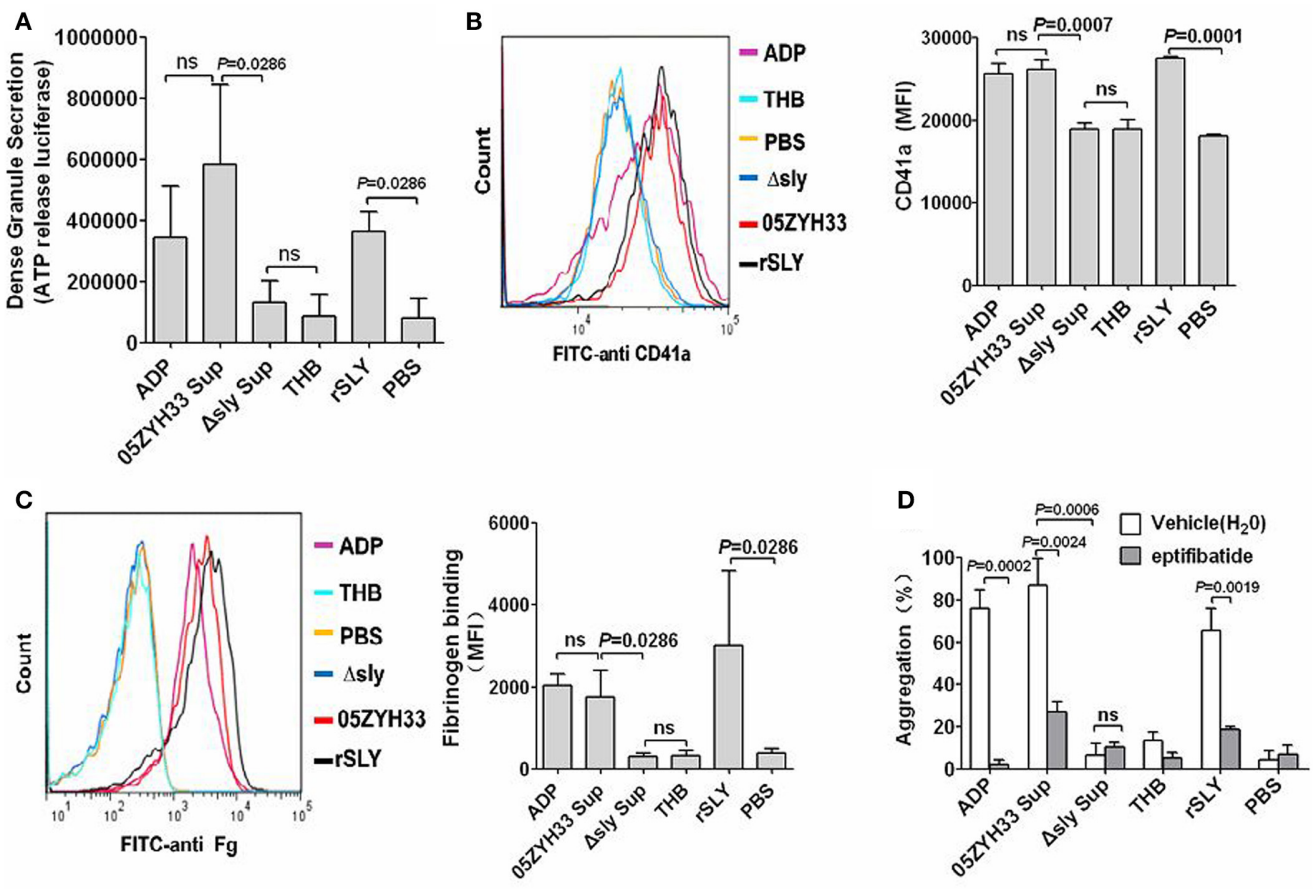
**Platelet activation by SLY, and GPIIb/IIIa (CD41a) mediated the SLY-induced platelet aggregation. (A)** SLY induces dense granule release from platelets. PRP was incubated with *S. suis* culture supernatant or SLY protein (1 μg/ml). The Mann–Whitney U-test was used for statistical analysis. **(B,C)** *S. suis* culture supernatant or rSLY protein (1 μg/ml)-induced surface GPIIb/IIIa (CD41a) increase and fibrinogen binding to platelets in human blood was assessed by flow cytometry (Methods Section). Representative histograms for the MFI of CD41a/fibrinogen binding are shown in (**B**, left panel). Unpaired two-tailed Student's *t*-test was used for **(B)** statistical analysis. Mann–Whitney U-test was used for **(C)** statistical analysis. **(D)** PRP was preincubated with eptifibatide (10 μM) for 15 min prior to addition of *S. suis* supernatant or SLY protein. Platelet aggregation was expressed as a final percentage of light transmission. Unpaired two-tailed Student's *t*-test was used for statistical analysis. ADP (20 μM) was used as the positive control for platelet activation. THB and PBS were the negative controls for the culture supernatant and proteins, respectively. Data in panels **(A–D)** are expressed as the mean ± *SD* of three independent experiments, with each experiment using blood from a different donor. *P* < 0.05 is considered to be the threshold for statistical significance; ns, not significant; 05ZYH33, wild type strain; Δsly, isogenic mutant of sly; Sup, supernatant; rSLY, recombinant SLY.

ATP exists in dense granules and is often used as a measure of dense granule release. Under resting conditions, platelets do not release ATP from their dense granules. 05ZYH33 supernatant, rSLY protein, or positive control ADP significantly stimulated ATP release from platelets (Figure 2A). GPIIb/IIIa is the most abundant platelet surface membrane glycoprotein (~70%), but there are additional pools of GPIIb/IIIa (~30%) in α-granule membranes (Phillips et al., 1988; Bennett, 2005), and the increased surface GPIIb/IIIa from the secreted contents of α-granules (when platelets activating) has also been detected by FITC conjugated anti-CD41a (GPIIb). Similarly to dense granules, 05ZYH33 supernatant and rSLY, as well as positive control ADP, increased the surface GPIIb/IIIa (CD41a, Figure 2B). However, the GPIIb/IIIa (CD41a) level stimulated by the supernatant from the Δsly group was almost equal to that of the THB/PBS control. Moreover, binding of the ligand of GPIIb/IIIa fibrinogen to the platelet surface also increased significantly in the presence of either 05ZYH33 supernatant, rSLY, or ADP (positive control); however, the level of fibrinogen upon platelets exposed to the supernatant from Δsly was as low as those of the background controls THB and PBS (Figure 2C). These results indicate that SLY is the sole *S. suis* protein that activates human platelets.

GPIIb/IIIa is the prominent adhesion receptor on platelets by virtue of its role in mediating platelet aggregation (Zarbock et al., 2007). Therefore, eptifibatide, a specific inhibitor of fibrinogen binding to the GPIIb/IIIa receptor, was used to explore the role of GPIIb/IIIa in the SLY-induced platelet aggregation. Pre-incubation of platelets with eptifibatide (10 μM) significantly decreased the platelet aggregation induced by the supernatant of 05ZYH33 or rSLY (Figure 2D). Besides GPIIb/IIIa, P-selectin is another important adhesion molecule that mediates neutrophil–platelet, platelet–platelet, and monocyte–platelet interactions (Gottschalk et al., 1995). Although SLY induced P-selectin release in α-granules (data not shown), in contrast to eptifibatide, the platelet aggregation induced by SLY remained unaffected by pretreatment with an anti-P-selectin antibody (Figure S3).

### SLY-induced platelet activation and aggregation depend on pore formation on platelets

**Figure 3:**
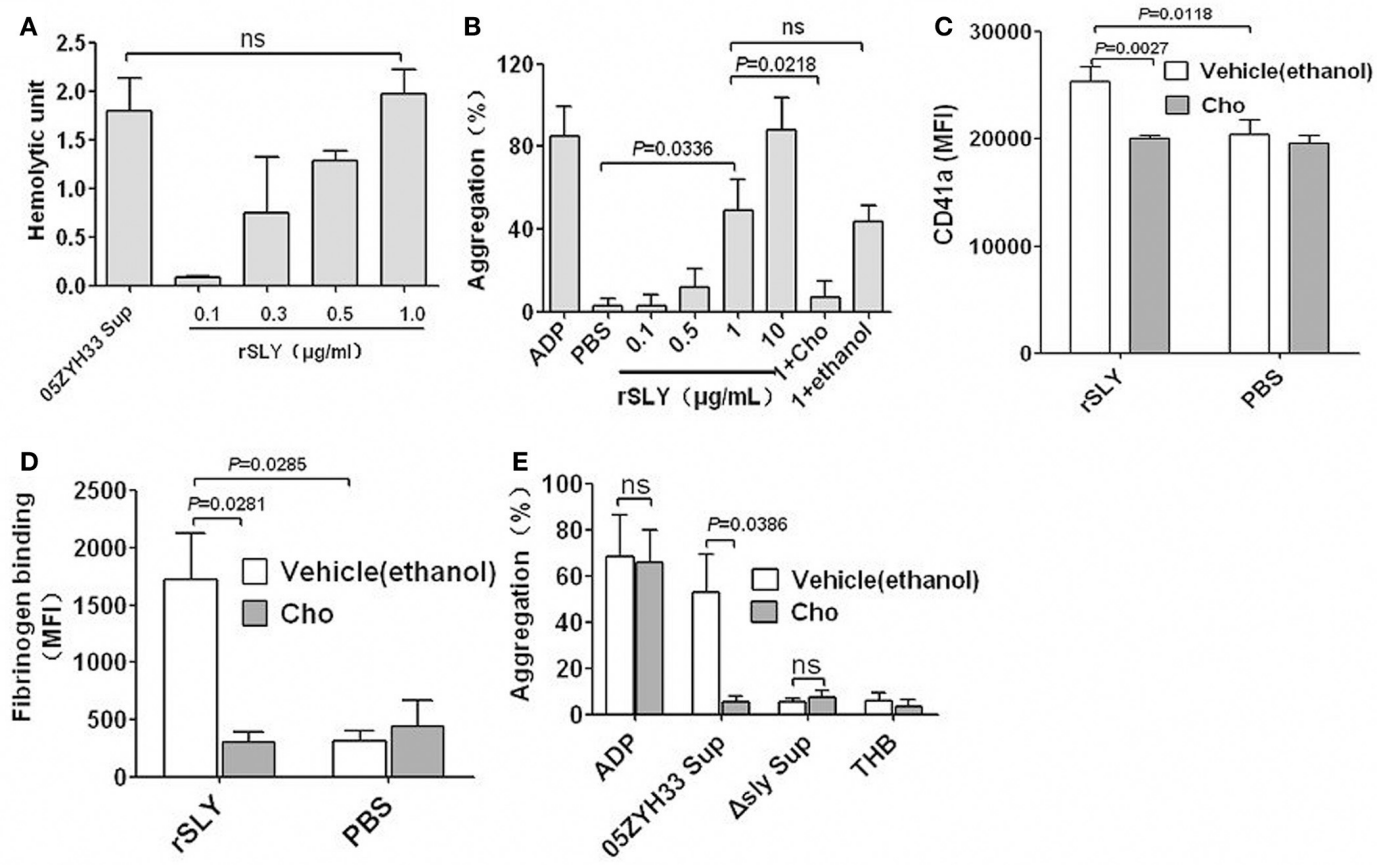
**SLY-induced platelet activation and aggregation dependent on pore formation on platelets. (A)** The hemolytic activity of 05ZYH33 supernatant and rSLY. Culture supernatants of *S. suis* 05ZYH33 and rSLY were tested the hemolytic activity as described by Materials and Methods. One hemolytic unit is defined as the reciprocal of the suilysin titer, which was calculated as the highest dilution of the supernatant/rSLY which caused at least 50% hemolysis. **(B)** Dose response of rSLY-induced platelet aggregation and the cholesterol inhibiting effect. **(C,D)** The cholesterol inhibiting effect of rSLY-induced surface GPIIb/IIIa (CD41a) increase and fibrinogen binding to platelets in human blood were assessed by flow cytometry (Methods Section). A total of 1 μg/ml of rSLY and 100 μg/ml of cholesterol were used. **(E)** The cholesterol (100 μg/ml) effect on *S. suis* supernatant-induced platelet aggregation was detected using a platelet aggregometer. Unpaired two-tailed Student's *t*-test was used for **(C)** statistical analysis. Unpaired *t*-test with Welch's correction was used for **(B,D,E)** statistical analysis. THB and PBS were the negative controls for culture supernatant and proteins, respectively. Cholesterol was dissolved in ethanol. rSLY, recombinant SLY; Cho, cholesterol; 1+Cho, 1 μg/ml of rSLY added to cholesterol. Data in panels **(A–E)** are expressed as the mean ± *SD* of three independent experiments, with each experiment using blood from a different donor. *P* < 0.05 is considered to be the threshold for statistical significance; ns, not significant; 05ZYH33, wild type strain; Δsly, isogenic mutant of sly; Sup, supernatant.

CDCs can bind to membrane cholesterol to create large pores (350–450 Å in diameter) and consequently lyse the target cells (Rossjohn et al., 1997; Gilbert et al., 1999). Free cholesterol can inhibit pore formation by CDCs (Giddings et al., 2004; Xu et al., 2010). To determine whether SLY-induced platelet aggregation is caused by pore formation, the cholesterol inhibition assay was used in this study. One μg/ml rSLY exhibited equal hemolytic activity to the supernatant of 05ZYH33 used in this study (Figure 3A). Substantial platelet aggregation occurred when rSLY was 1 μg/ml, whereas 0.1 μg/ml rSLY with lowest hemolytic activity did not (Figure 3B). Interestingly, cholesterol (100 μg/ml) that inhibited the hemolytic activity of rSLY (Bi et al., 2015) abrogated platelet aggregation (Figures 3B). ADP (20 μM) and rSLY (10 μg/ml) induced 93.9% platelet aggregation without a lag time, whereas at a low dose of rSLY (1 μg/ml), there was an approximate 3-min lag time prior to massive stimulation (Figure S4), possibly because of SLY (10 μg/ml)-induced platelet lysis leading to the highest light transmission. As expected, cholesterol (100 μg/ml) significantly inhibited rSLY (1 μg/ml)-induced increased surface GPIIb/IIIa (CD41a) and fibrinogen binding (Figures 3C,D). Cholesterol subsequently inhibited 05ZYH33 supernatant-induced platelet aggregation (Figure 3E). These results suggest that SLY-induced platelet activation and aggregation depend on pore formation on platelets.

### SLY-induced platelet activation and aggregation required pore formation-dependent Ca^2+^ influx

**Figure 4:**
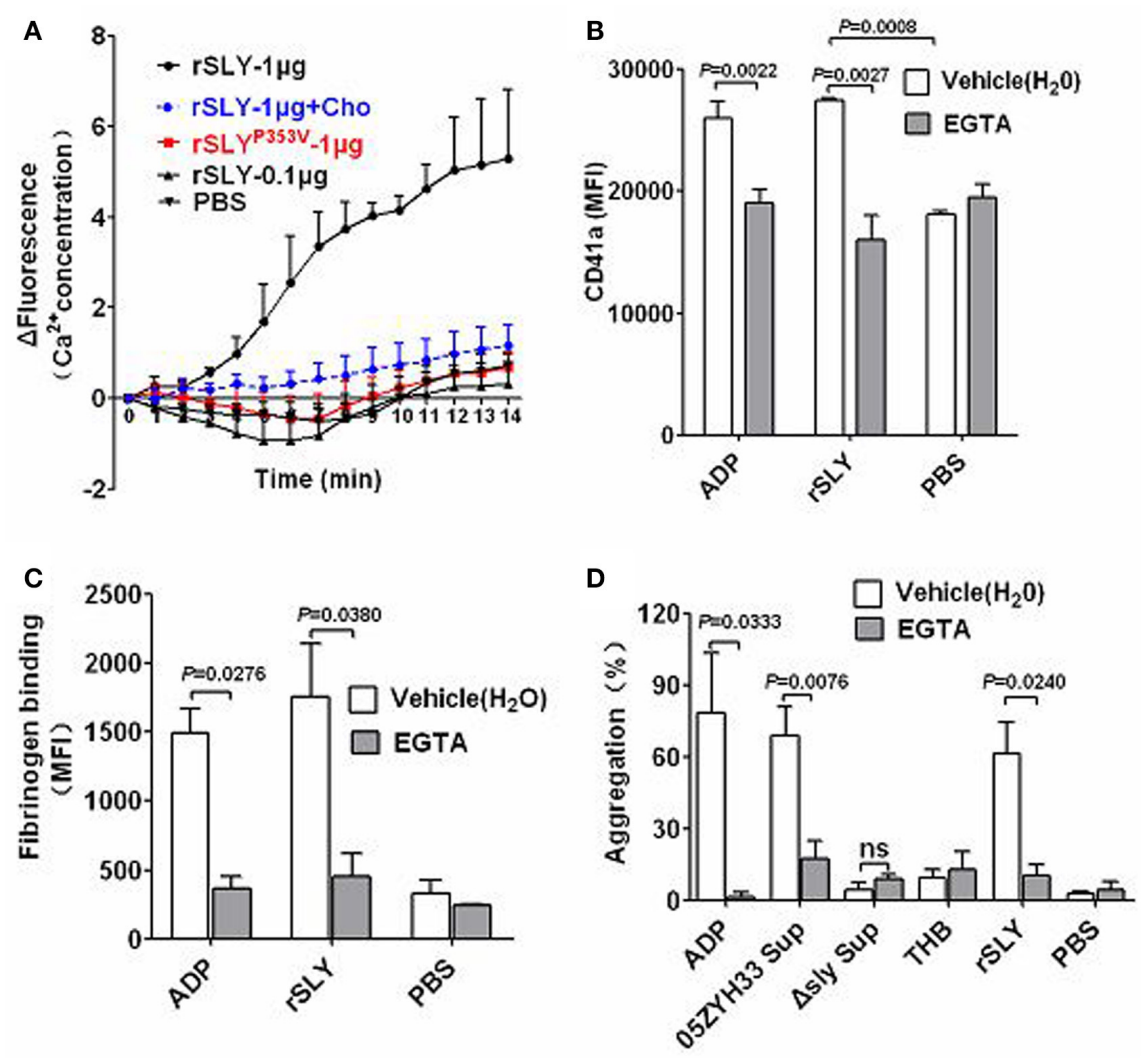
**Pore dependent Ca^**2+**^ influx by SLY triggers platelet activation and aggregation. (A)** rSLY induces Ca^2+^ influx in human platelets. The purified platelets marked with fluo-8 were resuspended in HBSS (with 2 mM Ca^2+^), and rSLY (1 μg/ml), rSLY (0.1 μg/ml), rSLY^P353V^ (1 μg/ml), cholesterol (10 μg/ml), or other control reagents were added. Ca^2+^ influx to platelets was detected using a Varioskan Flash Multiplate Reader. **(B,C)** The EGTA effect on rSLY-induced surface GPIIb/IIIa (CD41a) increase and fibrinogen binding to platelets in human blood was assessed by flow cytometry (Methods Section). Human blood was preincubated with EGTA (3 mM) for 10 min prior to addition of rSLY (1 μg/ml). Unpaired two-tailed Student's *t*-test was used for **(B)** statistical analysis. **(D)** The EGTA (3 mM) effect on *S. suis* supernatant-induced platelet aggregation was detected using a platelet aggregometer (Methods Section). Unpaired *t*-test with Welch's correction was used for **(C,D)** statistical analysis. THB and PBS were the negative controls for culture supernatant and proteins, respectively. EGTA was dissolved in H_2_O. Data in panels **(A–D)** are expressed as the mean ± *SD* of three independent experiments, with each experiment using blood from a different donor. *P* < 0.05 is considered to be the threshold for statistical significance; ns, not significant; Cho, cholesterol; rSLY, recombinant SLY; rSLY^P353V^, recombinant non-hemolytic mutant of SLY^P353V^; 05ZYH33, wild type strain; Δsly, isogenic mutant of sly; Sup, supernatant.

A previous study reported that *Staphylococcus aureus* α-toxin promotes assembly of the prothrombinase complex, and that this process was dependent on Ca^2+^ but not on platelet lysis (Arvand et al., 1990). However, α-toxin belongs to the small β-barrel pore-forming group of toxins with different structures to other CDCs (Meesters et al., 2009; Xu et al., 2010). The mechanisms underlying SLY-induced Ca^2+^ influx were investigated in the present study. In Ca^2+^-free HBSS, rSLY (1 μg/ml) caused a mild increase in the intracellular Ca^2+^ levels in platelets (data not shown). In contrast, in HBSS containing 2 mM Ca^2+^, 1 μg/ml of SLY increased platelet intracellular Ca^2+^ levels dramatically, whereas 0.1 μg per mL SLY and the non-hemolytic recombinant mutant SLY^P353V^ did not (Figure 4A). In addition, cholesterol (100 μg/ml) strongly diminished rSLY-induced Ca^2+^ influx (Figure 4A). These results indicate that rSLY-induced Ca^2+^ influx may depend on rSLY cytotoxicity.

Agonists that stimulate platelet aggregation (e.g., ADP and platelet-activating factor, PAF) commonly cause platelets to mobilize Ca^2+^ stores and take up extracellular Ca^2+^, thereby increasing the concentration of cytosolic Ca^2+^ (Bird et al., 2004). The roles of Ca^2+^ influx in platelet activation and aggregation were studied further by using EGTA to chelate the extracellular Ca^2+^. Blockage of Ca^2+^ influx by EGTA significantly reduced rSLY-induced platelet surface GPIIb/IIIa (CD41a, Figure 4B) and fibrinogen binding (Figure 4C). Consequently, EGTA also inhibited SLY-induced platelet aggregation (Figure 4D). With the ADP positive control, ADP-induced platelet aggregation was also blocked by EGTA (Figure 4D). Taken together, these results indicate that pore dependent Ca^2+^ influx plays essential roles in platelet activation and aggregation induced by SLY.

### Platelet signaling in response to SLY

PLC-β-IP3/DAG-MLCK (Gottschalk et al., 1995; Rebecchi and Pentyala, 2000; Rhee, 2001) and Rho-ROCK-MLCK signaling (Klages et al., 1999) are usually involved in platelet activation by agonists such as ADP and PAF. Signaling by PLC-β-IP3/DAG-MLCK is often accompanied by an increase in intracellular Ca^2+^ binding to calmodulin (Bird et al., 2004). The final signaling events for the pathways described above involve phosphorylation of MLC by MLCK and this leads to actin–myosin interactions, the consequence of which is platelet degranulation (Gottschalk et al., 1995).

**Figure 5:**
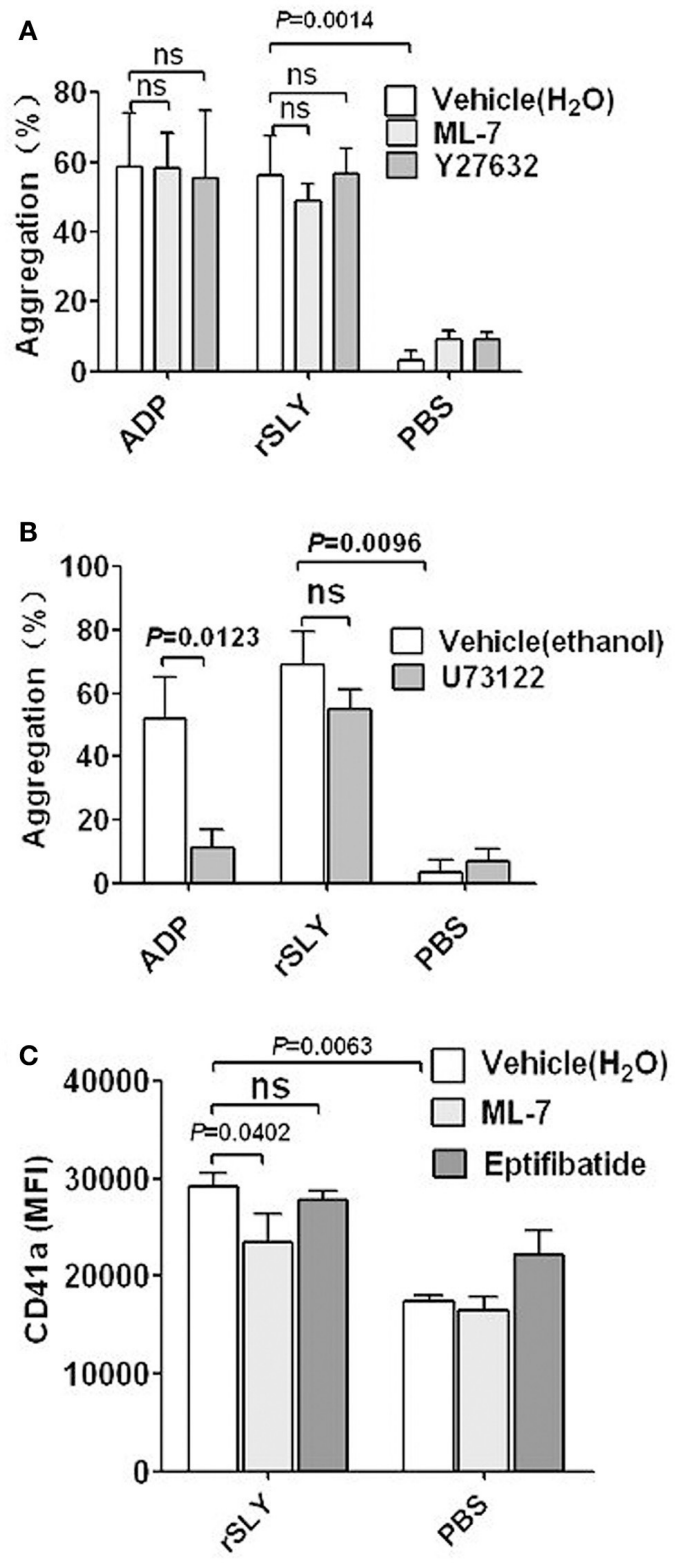
**Platelet signaling in response to SLY. (A,B)** rSLY (1 μg/mL)-induced platelet aggregation in PRP pretreated with or without MLCK inhibitor ML-7 (100 μM), ROCK inhibitor Y27632 (100 μM), and PLC-β inhibitor U73122 (20 μM). Unpaired *t*-test with Welch's correction was used for **(A,B)** statistical analyses. **(C)** The effects of ML-7 (100 μM) and eptifibatide (10 μM) on rSLY-induced surface GPIIb/IIIa (CD41a) increase. Unpaired two-tailed Student's *t*-test was used for statistical analysis. PBS is the negative control for rSLY protein. ML-7, Y27632, and eptifibatide were dissolved in H_2_O. U73122 was dissolved in ethanol. Data in panels **(A–C)** are given as the mean ± *SD* of 3–8 independent experiments from different blood donors. *P* < 0.05 is considered to be the threshold for statistical significance; ns, not significant.

To investigate the possible signaling involved in platelet activation induced by SLY, specific inhibitors were used. Both the MLCK-specific inhibitor ML-7 and ROCK-specific inhibitor Y27632 had no effect on SLY or ADP-induced platelet aggregation (Figure 5A). However, ML-7 had some effects on the increased surface GPIIb/IIIa (CD41a) that from α-granules release (Figure 5C). In combination with the results shown in Figure 2D, these results suggest that SLY-induced aggregation seems to depend on the high-affinity of fibrinogen for activated GPIIb/IIIa (CD41a), rather than increase the number of GPIIb/IIIa (~30%) resulting from α-granule release induced by SLY (Figure 3C).

The PLC-β-specific inhibitor U73122 significantly decreased ADP-induced platelet aggregation, but had no effect on SLY-induced platelet aggregation (Figure 5B). Interestingly, these data suggest that SLY-induced platelet aggregation did not occur via PLC-β-IP3/DAG-MLCK or Rho-ROCK-MLCK signaling.

SLY activates human platelets resulting in an “inside-out” signaling leading to integrin GPIIb/IIIa (CD41a) activation and this promotes binding to the fibrinogen present in the plasma (Figure 2B). The ligand-occupied GPIIb/IIIa (CD41a) in turn usually reinforces activation of “outside-in” signaling leading to secretion of α-granules. However, eptifibatide had no effect on GPIIb/IIIa (CD41a) (Figure 5C); this probably results from the limited amounts of GPIIb/IIIa (~30%) in the membranes of α-granule (Phillips et al., 1988; Bennett, 2005).

### Intravascular thrombosis and associated liver injury caused by SLY in mice

**Figure 6:**
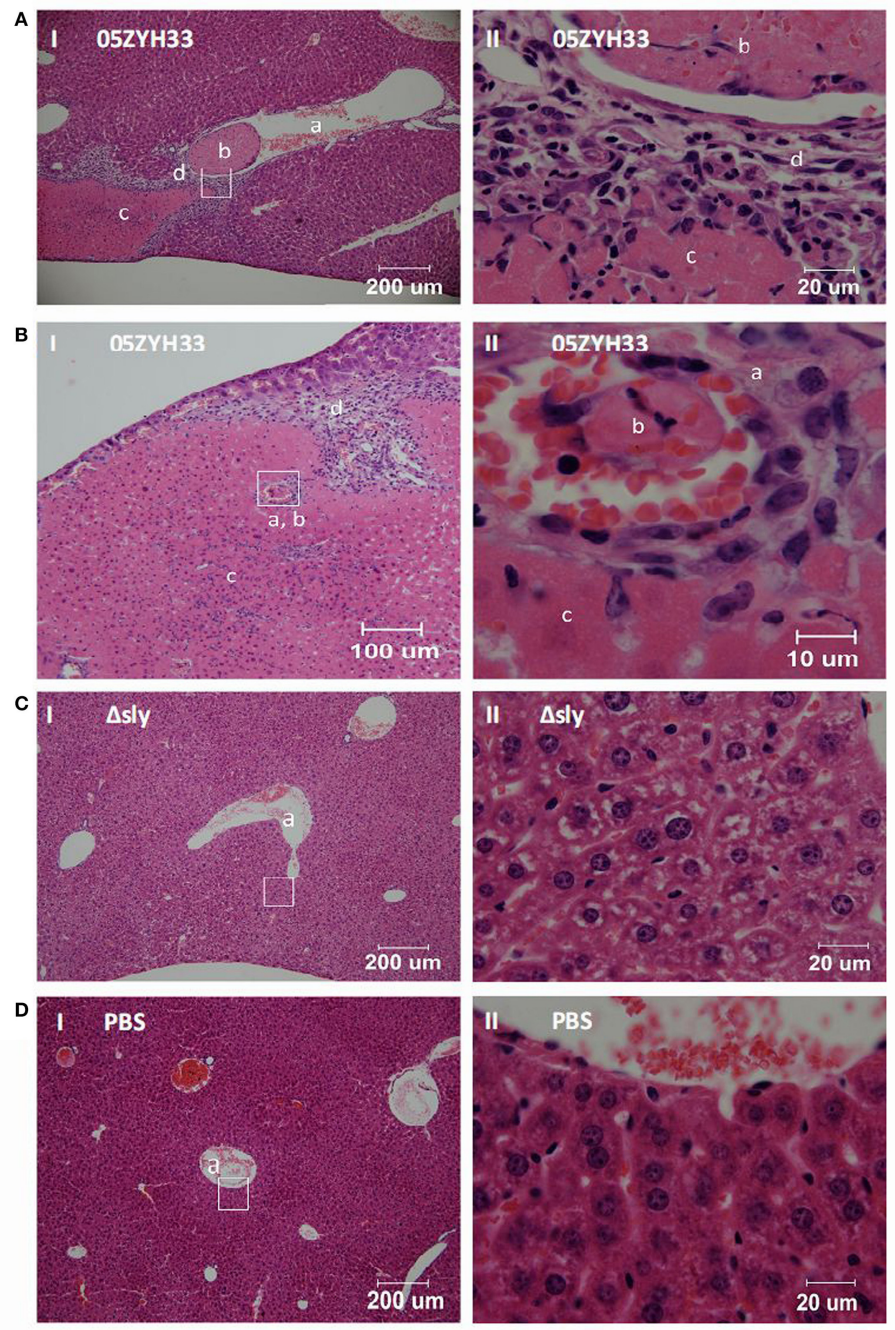
**SLY contributes to the intravascular thrombosis and its associated liver injury caused by ***S. suis*****. Female BALB/c mice (6–8 weeks old) were challenged with **(A,B)** 05ZYH33; **(C)** mutant Δsly (~1 × 10^8^ CFU); **(D)** PBS control through the caudal vein. Pathological changes were observed by hematoxylin and eosin pathological staining of tissue sections at 48 h post-inoculation. Panels numbered **II** are the enlarged images from the white boxes in panels numbered **I**. **a, b, c**, and **d** in the panels represent blood vessels, intravascular thrombosis, coagulative necrosis, and leukocyte infiltration, respectively.

In our early study, *S. suis* 05ZYH33 induced higher mean platelet volume and lower platelets counts in mice blood than *S. suis* Δsly, suggesting that SLY induced platelet activation in mice-infecting model (Zhang et al., 2016). Moreover, the contribution of SLY to platelet aggregation in this study led us to further examine the role of simultaneous targeting of platelets in relation to pathology *in vivo*. Female BALB/c mice (6–8 weeks old) were challenged with 05ZYH33 or mutant Δsly bacteria, through the caudal vein. Pathological changes in the mice were observed by hematoxylin and eosin stained tissue sections at 48 h post-inoculation. Vessels within the liver tissues infected by 05ZYH33 showed a remarkable level of intravascular thrombi (Figures 6A-b,B-b). Moreover, coagulative necrosis (Figures 6A-c,B-c) and leukocyte infiltration (Figures 6A-d,B-d) surrounding the vein vessel (Figure 6A-a) or artery vessel (Figure 6B-a) were apparent. However, intravascular thrombi with surrounding coagulative necrosis and leukocyte infiltration were not observed in the vessels of live tissues infected by Δsly (Figure 6C) or in the PBS control group (Figure 6D). Part II panels represent the enlarged images of the white boxes shown in part I panels of Figures 6A–D.

### Other CDCs promote the platelet aggregation required for pore dependent calcium influx

**Figure 7:**
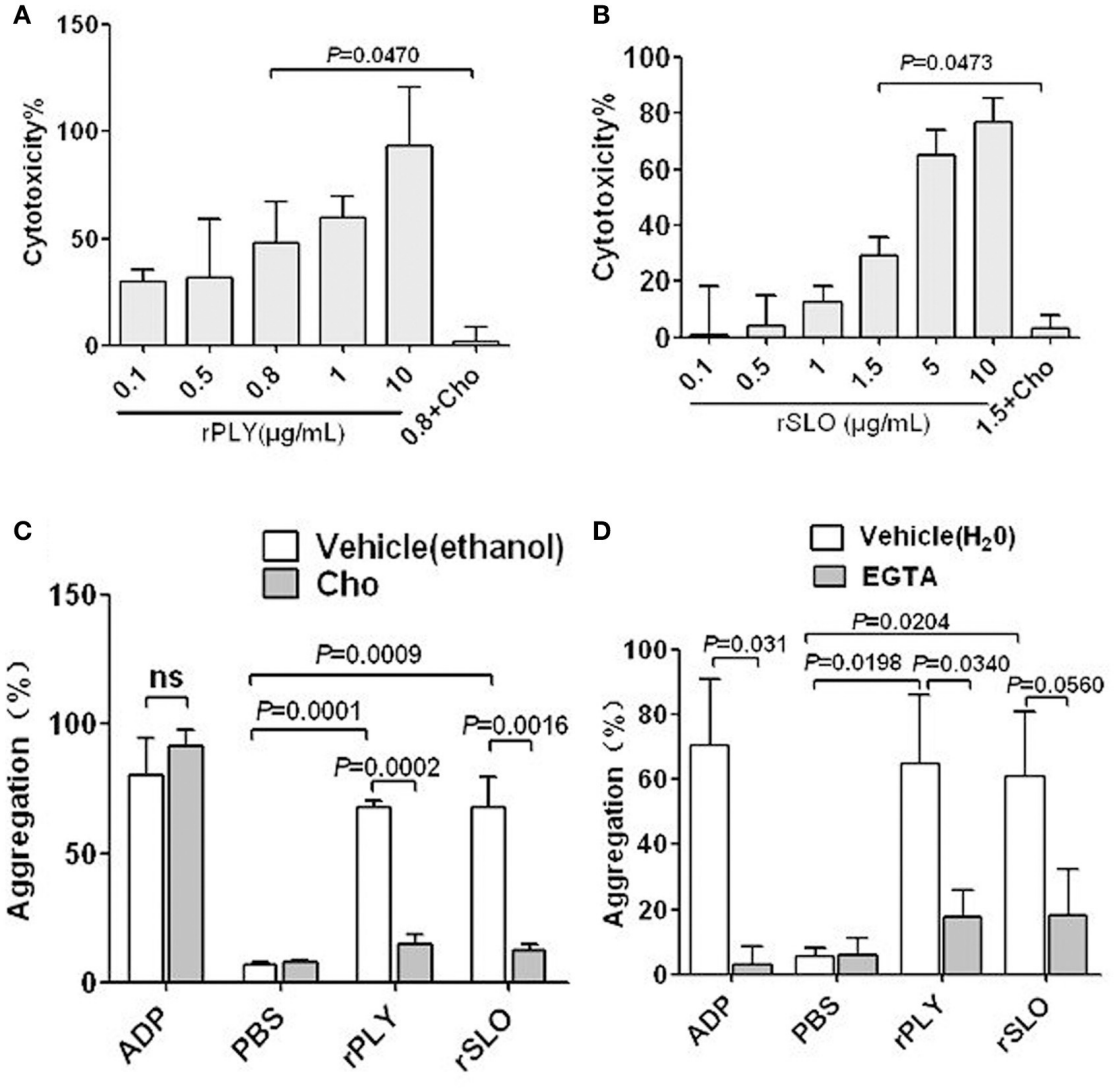
**rPLY and rSLO induce platelet aggregation via pore dependent calcium influx**. The cytotoxicity of rPLY **(A)** and rSLO **(B)** against platelets and the cholesterol inhibiting effect were assessed by an LDH assay (Methods Section). **(C)** The cholesterol (100 μg/mL) effect on rPLY (0.8 μg/mL)- and rSLO (1.5 μg/mL)-induced platelet aggregation. **(D)** The EGTA (3 mM) effect on rPLY (0.8 μg/mL)- and rSLO (1.5 μg/mL)-induced PNA formation. PBS acted as the negative control for the recombinant proteins. Cholesterol and EGTA were dissolved in ethanol and H_2_O, respectively. Unpaired *t*-test with Welch's correction was used for **(A,B,D)** statistical analyses. Unpaired two-tailed Student's *t*-test was used for **(C)** statistical analysis. Data in panels **(A–D)** are given as the mean ± *SD* of three independent experiments, with each experiment using blood from a different donor. *P* < 0.05 is considered to be the threshold for statistical significance; ns, not significant; rPLY, recombinant pneumolysin; rSLO, recombinant streptolysin O; Cho, cholesterol; 0.8+Cho, 0.8 μg/mL of rPLY added to cholesterol; 1.5+Cho, 1.5 μg/mL of rSLO added to cholesterol.

PLY and SLO are important pathogen toxins of *S. pneumoniae* and *S. pyogenes*, respectively. They are toxins from group I, which comprises typical CDCs with high affinity for cholesterol and high structural similarity to SLY (Giddings et al., 2004). Therefore, PLY and SLO possibly share similar functional characteristics to SLY. Interestingly, cholesterol completely inhibits the cytotoxicity of 0.8 μg/ml PLY and 1.5 μg/ml SLO (Figures 7A,B), and also abrogates their platelet aggregation inducing abilities (Figure 7C). Moreover, EGTA has inhibiting effects on the platelet aggregation induced by PLY and SLO (Figure 7D), although the difference was not statistically significant in the SLO group. It is possible that CDC-mediated platelet aggregation is a common mechanism used by a wide range of bacteria, and is triggered by pore dependent calcium influx.

## Discussion

We have identified the cytolysin SLY is the main stimulus required for human platelet activation in *S. suis* 05ZYH33, which is a sequenced strain belonging to sequence type 7 (ST-7) strains that caused the 2005 *S. suis* outbreak and STSS in China (Ye et al., 2009). Our systematic analysis of SLY found that it induces platelet activation and aggregation triggered by pore dependent Ca^2+^ influx.

Different from *S. aureus, S. pyogenes*, and *S. pneumoniae*, SLY was found to be the sole factor inducing human platelet activation and aggregation. For example, the fibrinogen-binding proteins, ClfA, ClfB, and SdrE of *S. aureus* can all interact with GPIIb/IIIa, generating an outside-in signal to trigger platelet aggregation (O'brien et al., 2002; Liu et al., 2007). *S. suis* is an emerging human pathogen that causes STSS. ST-7 strains caused the outbreak in humans in China in 2005 and were more toxic to human peripheral blood mononuclear cells than ST-1 strains (mainly referring to the European virulent strains) (Ye et al., 2006). In particular, we have previously shown that the ST-7 strains produce more SLY protein than the non-epidemic strains, and this contributes to invasive infections (He et al., 2014). Additionally, similar to STSS patients infected by *S. suis*, 05ZYH33 induced intravascular thrombosis and associated-liver injury in our mouse infection model (Figure 6). Therefore, SLY may be a potential therapeutic target for preventing *S. suis*-mediated platelet activation, thrombocytopenia related DIC, and purpura gangrenosa.

Although it has been reported that there are 20 types of bacterial cytolysin belonging to Group I CDCs with high affinity to cholesterol (Tabata et al., 2014), there is scant evidence showing associations existing between Group I CDCs and platelet activation. Ohkuni et al. has reported that recombinant SLY, PLY, and Sm-hPAF were stimuli capable of inducing platelet aggregation (Ohkuni et al., 2012). However, the mechanisms underlying how these bacterial toxins induce platelet activation are not clearly understood. In a comprehensive review, Cox et al. proposed that pore-forming toxins activate platelets in a manner similar to α-toxin (Cox et al., 2011), but the data in the articles cited in this review did not clearly support this viewpoint. Our current study presents direct evidence showing that SLY induced a pore dependent Ca^2+^ influx in platelets, which was required for SLY-induced platelet aggregation; the other two CDCs, PLY, and SLO, also induced platelet aggregation in a similar manner.

The present study also found that SLY-induced platelet GPIIb/IIIa (CD41a) activation was Ca^2+^ influx dependent, suggesting that stimulation of this adhesion factor appears to be downstream of SLY-induced Ca^2+^ influx. Contrastingly, MLCK may not be involved in platelet aggregation because ML-7 failed to suppress SLY-induced platelet aggregation although it inhibited GPIIb/IIIa (CD41a) release in α-granules (Figures 5A,C). SLY-induced Ca^2+^ influx may directly activate “inside-out” signaling to enhance the affinity of GPIIb/IIIa to fibrinogen, which subsequently induces platelet aggregation.

We also found that the PLC-β inhibitor U73122 dramatically decreased ADP-induced platelet aggregation but had no impact on SLY-induced platelet aggregation. The data imply that the signaling events involved in SLY-induced platelet activation may differ from typical platelet agonists such as ADP and thromboxane A2 (TXA2) because these are dependent on PLC-β associated signaling (Zarbock et al., 2007). Furthermore, Born aggregometry revealed that SLY-induced platelet aggregation showed a 3-min lag time prior to massive aggregation occurring (Figure S4). In contrast, most platelet agonists such as ADP cause rapid activation (Cox et al., 2011). The differential dynamics for SLY-induced platelet aggregation and ADP-induced platelet aggregation also suggest that distinct signaling might be induced by SLY.

**Figure 8:**
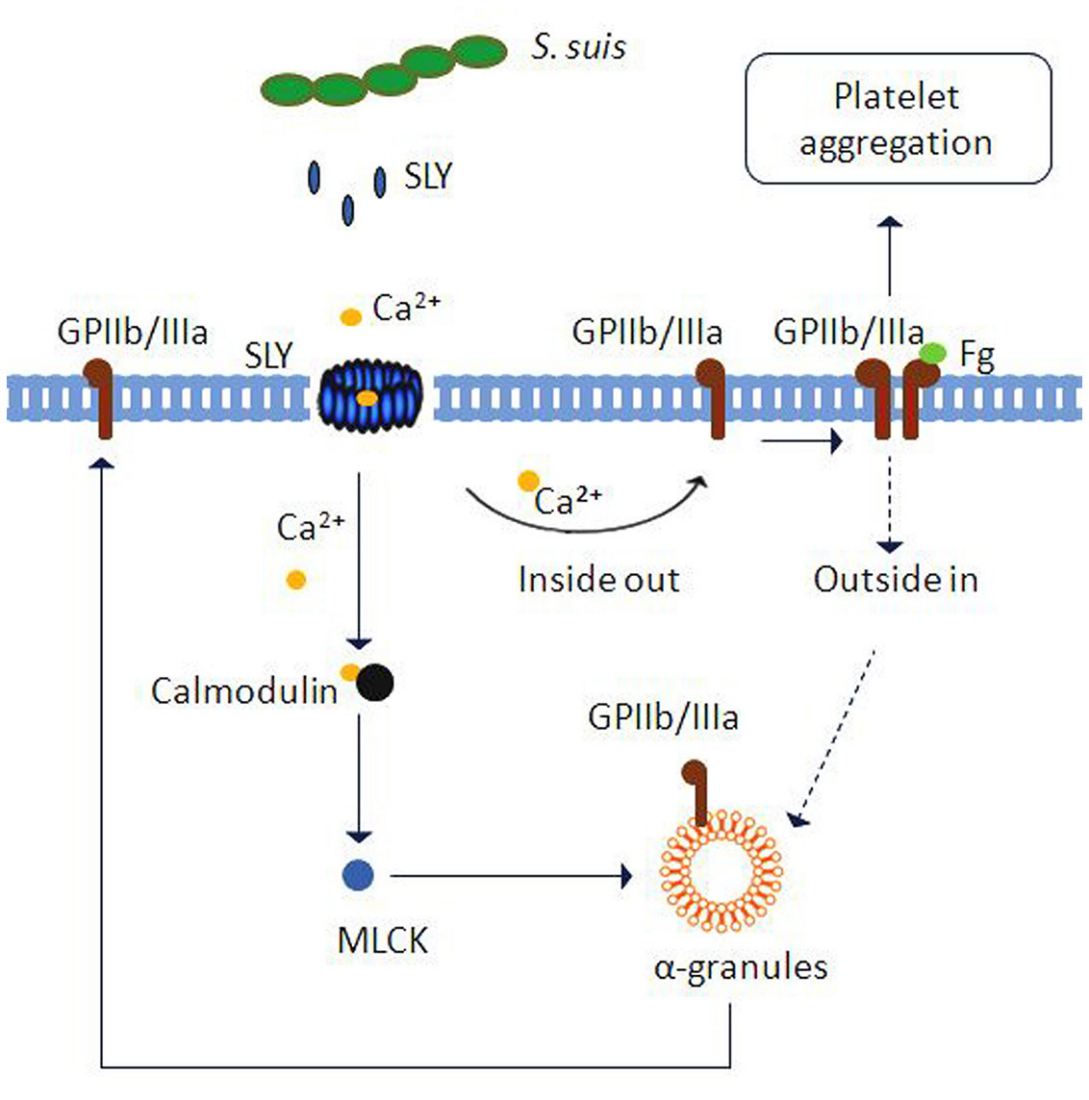
**Schematic representation of platelet activation and aggregation induced by ***S. suis*****. Ca^2+^ influx across transmembrane pores created by SLY, the CDC of *S. suis*, can trigger inside-out signaling leading to integrin GPIIb/IIIa activation and α-granule (GPIIb/IIIa) or dense granule (ATP) secretion. Subsequently, GPIIb/IIIa activation leads to platelet aggregation. Additionally, Ca^2+^-MLCK signaling is involved in α-granule release induced by SLY.

Overall, we found that SLY, which was secreted by *S. suis* 05ZYH33 at the stationary growth phase, stimulated platelet activation and aggregation. This stimulation required SLY-induced Ca^2+^ influx and subsequent “inside-out” signaling to activate GPIIb/IIIa (CD41a) (as proposed in Figure 8). Moreover, PLY, SLO, and SLY, which are all Group I CDCs, seem to share similar mechanisms for inducing platelet aggregation. We foresee that this similar mode of activation identifies Group I CDCs as potential therapeutic targets for preventing bacterial-induced platelet activation and thrombotic-related disorders.

## Author contributions

YY, SZ, and YJ conceived and designed the experiments. SZ, SC, and JW performed the experiments. SZ, JY, ZP, and YY analyzed the data. KL, LL, and YZ contributed reagents/materials/analysis tools. YY wrote the paper. All authors contributed to the interpretation of the data and writing of the manuscript and read and approved the final version.

## Funding

This work was supported by grants from the National Basic Research Program (973) of China (2012CB518804) and the National Natural Science Foundation of China (81371766).

### Conflict of interest statement

The authors declare that the research was conducted in the absence of any commercial or financial relationships that could be construed as a potential conflict of interest.

## Supplementary material

Figure S1 **The purity of human platelets in PRP. (A)** Leukocytes in human blood were analyzed by flow cytometry as forward- and side-scatter. **(B)** PRP was analyzed by flow cytometry as forward- and side-scatter. **(C)** The percent of CD41a positive cells in panel **(B)** was shown as representative histograms. The platelets in PRP were detected by flow cytometry analysis using FITC conjugated anti-CD41a (clone HIP8) antibody.

Figure S2 **The activity of ***S. suis***-induced platelet aggregation. (A)** The culture supernatant and the washed bacteria cells of *S. suis* were added to PRP in a stirred cuvette. Platelet aggregation was expressed as a final percentage of light transmission detected by Platelet Aggregometer se-2000. ADP (20 μM) and THB were used as the positive controls and negative controls, respectively. Data are expressed as the mean ± *SD* of three independent experiments, with each experiment using blood from a different donor. **(B)** The platelet aggregation curves shown in panel B are from one representative experiment of three independent experiments. *S. suis* 4 (SLY^+^, 89K^+^) isolated from human patient in China (2005); *S. suis* 4005 (SLY^+^, 89K^−^) and s735 (SLY^+^, 89K^−^) isolated from diseased piglets in Netherlands; *S. suis* 1940 (SLY^+^, 89K^−^) isolated from diseased piglets in China (1980); Sup, supernatant; SLY, suilysin; 89K^−^, 89 kb pathogenicity island.

Figure S3 **S. s***uis*** supernatant and rSLY-induced platelet aggregation in PRP was assessed in the presence of 15 μL anti-CD62P blocking antibody or an isotype-matched control antibody (BD Bioscience)**. Unpaired two-tailed Student's *t*-test was used for statistical analysis. Data in are expressed as the mean ± *SD* for three independent experiments, with each experiment using blood from a different donor. *P* < 0.05 is considered as the threshold for significance; ns, no significance; 05ZYH33, wild type strain; Δsly, The isogenic mutants of sly; Sup, supernatant.

Figure S4 **Dose response of rSLY-induced platelet aggregation**. Serial concentrations of SLY were added to platelet-rich plasma (PRP) in a stirred cuvette. Platelet aggregation was expressed as a final percentage of light transmission.

Table S1 **Bacterial strains and plasmids used in this study**.
