# Supplementary figures and images for: Effects of Suilysin on Streptococcus suis-Induced Platelet Aggregation
Source: Front Cell Infect Microbiol. 2016 Oct 17;6:128. doi: 10.3389/fcimb.2016.00128 (PMC5065993; doi:10.3389/fcimb.2016.00128)

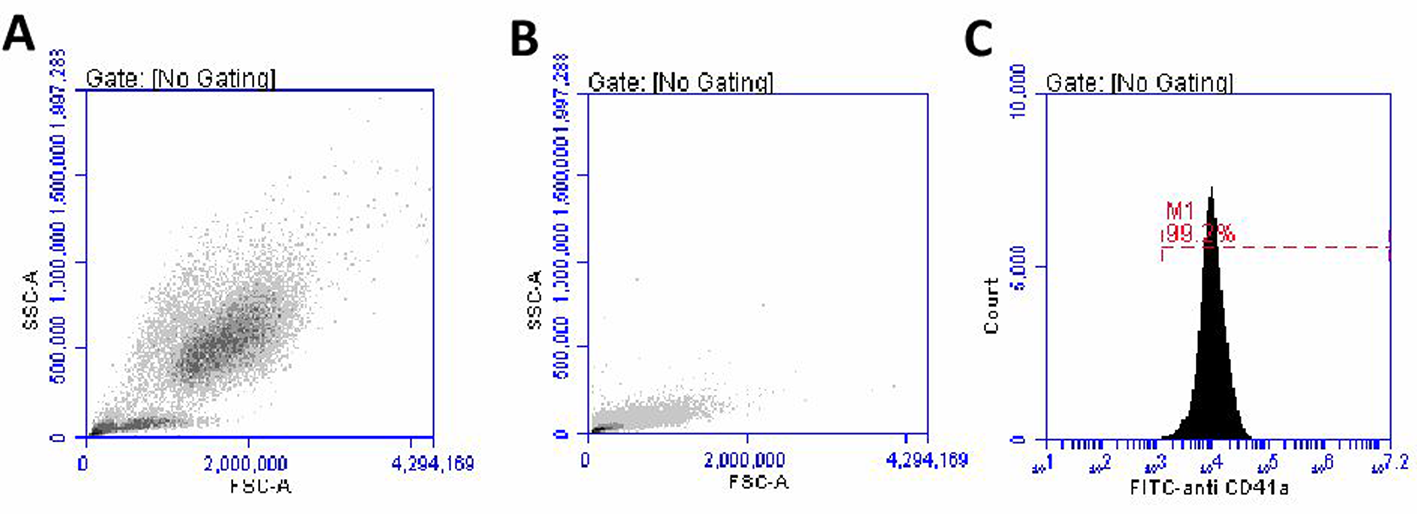

Supplement: Figure S1 — The purity of human platelets in PRP. (A) Leukocytes in human blood were analyzed by flow cytometry as forward- and side-scatter. (B) PRP was analyzed by flow cytometry as forward- and side-scatter. (C) The percent of CD41a positive cells in panel (B) was shown as representative histograms. The platelets in PRP were detected by flow cytometry analysis using FITC conjugated anti-CD41a (clone HIP8) antibody. [file Image1.TIF]

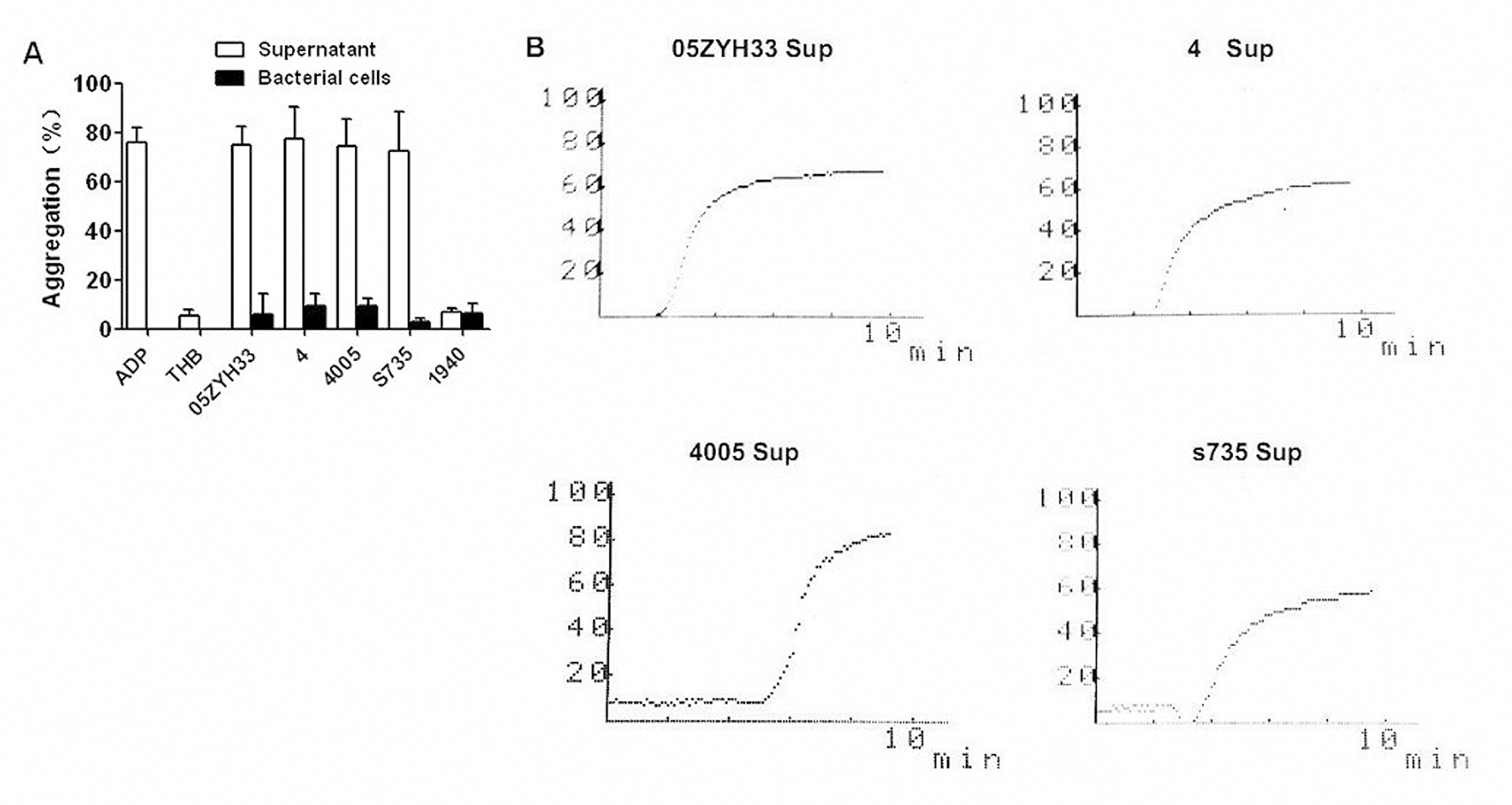

Supplement: Figure S2 — The activity of S. suis-induced platelet aggregation. (A) The culture supernatant and the washed bacteria cells of S. suis were added to PRP in a stirred cuvette. Platelet aggregation was expressed as a final percentage of light transmission detected by Platelet Aggregometer se-2000. ADP (20 μM) and THB were used as the positive controls and negative controls, respectively. Data are expressed as the mean ± SD of three independent experiments, with each experiment using blood from a different donor. (B) The platelet aggregation curves shown in panel B are from one representative experiment of three independent experiments. S. suis 4 (SLY+, 89K+) isolated from human patient in China (2005); S. suis 4005 (SLY+, 89K−) and s735 (SLY+, 89K−) isolated from diseased piglets in Netherlands; S. suis 1940 (SLY+, 89K−) isolated from diseased piglets in China (1980); Sup, supernatant; SLY, suilysin; 89K−, 89 kb pathogenicity island. [file Image2.TIF]

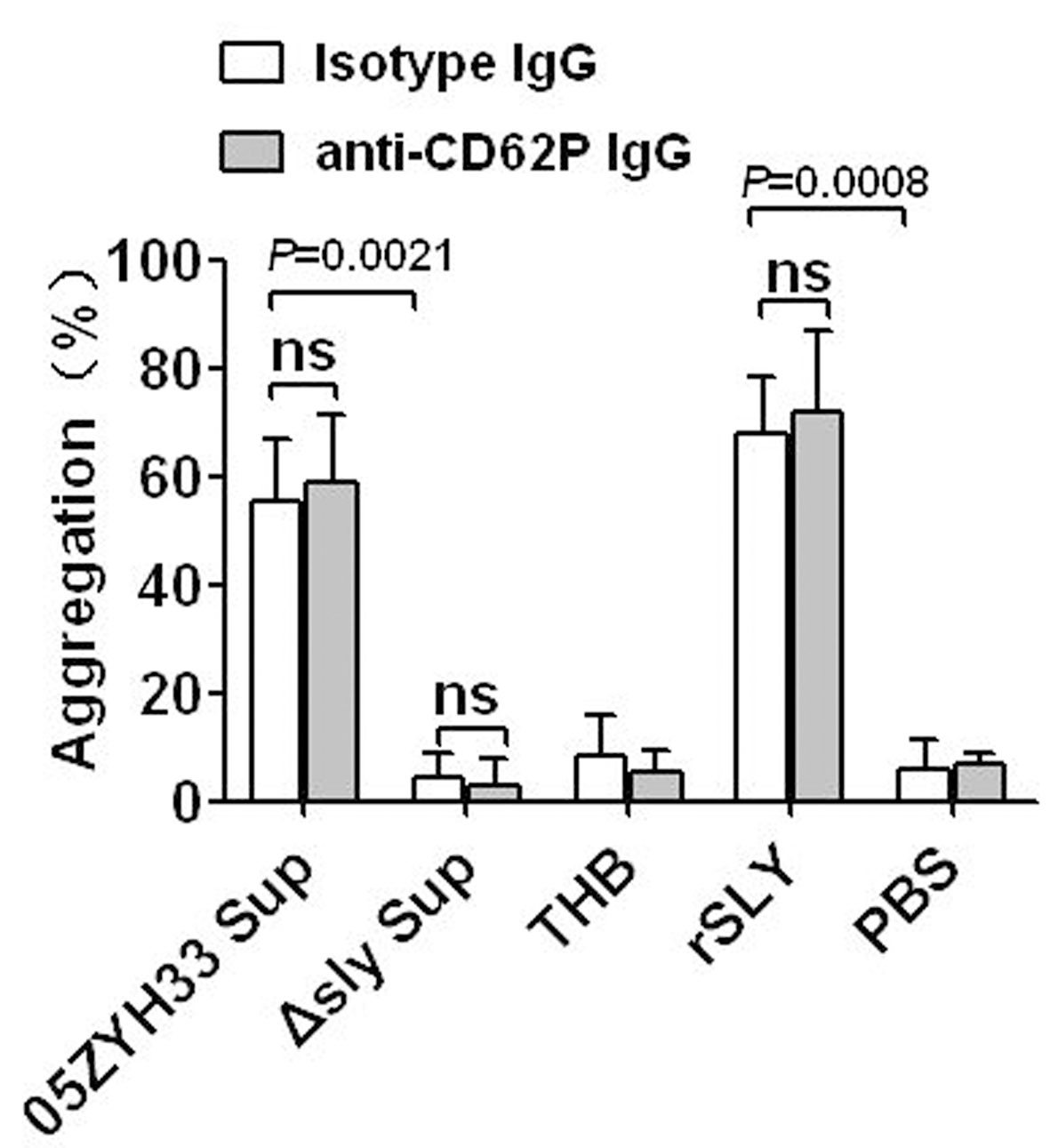

Supplement: Figure S3 — S. suis supernatant and rSLY-induced platelet aggregation in PRP was assessed in the presence of 15 μL anti-CD62P blocking antibody or an isotype-matched control antibody (BD Bioscience). Unpaired two-tailed Student's t-test was used for statistical analysis. Data in are expressed as the mean ± SD for three independent experiments, with each experiment using blood from a different donor. P < 0.05 is considered as the threshold for significance; ns, no significance; 05ZYH33, wild type strain; Δsly, The isogenic mutants of sly; Sup, supernatant. [file Image3.TIF]

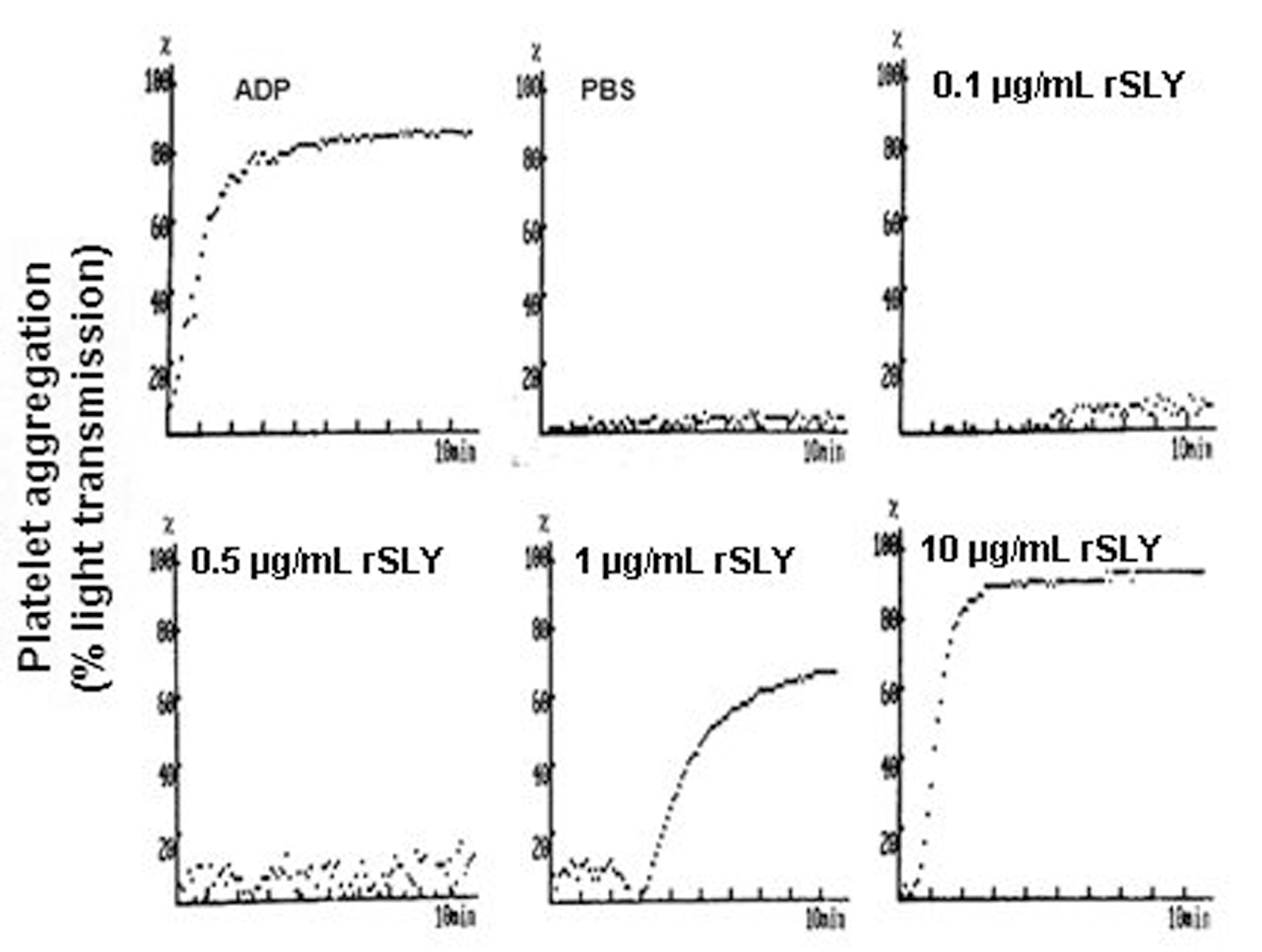

Supplement: Figure S4 — Dose response of rSLY-induced platelet aggregation. Serial concentrations of SLY were added to platelet-rich plasma (PRP) in a stirred cuvette. Platelet aggregation was expressed as a final percentage of light transmission. [file Image4.TIF]
