# Supplementary material for: Effects of Suilysin on Streptococcus suis-Induced Platelet Aggregation
Source: Front Cell Infect Microbiol. 2016 Oct 17;6:128. doi: 10.3389/fcimb.2016.00128 (PMC5065993; doi:10.3389/fcimb.2016.00128)
Supplement: Table S1 — Bacterial strains and plasmids used in this study. [file Table1.DOCX]

**Supplemental Table S1. Bacterial strains and plasmids used in this study.**

| Strain or plasmid | Description^a^ or Sequence | Source, PCR products |
| --- | --- | --- |
| Strains  *E. coli* DH5α  05ZYH33  Δsly  Δmrp  1330  GAS | Description  Host for cloning vector  Virulent Chinese *S. suis* serotype 2 isolate  Gene sly knockout mutant *S. suis* strain; Cm^R^  Gene mrp knockout mutant *S. suis* strain; Cm^R^  The Canadian avirulent *S. suis* strain  Group A streptococcus, the M1 type, E477 | In this lab  In this lab  In this lab  In this lab  donated by Prof. Marcelo Gottschalk  In this lab |
| Plasmids  pET-28a :: *sly*  pET-28a :: *Fhb*  pTrcHis  pTrcHis :: *ply*    pTrcHis :: *slo* | Description  pET-28a containing the *sly* ORF with removing its signal sequence; Kan^R^  pET-28a containing the *Fhb* ORF with removing its signal sequence; Kan^R^  Expression vector, Amp^R^  pTrcHis containing the ORF *ply* with removing its signal sequence; Amp^R^  pTrcHis containing the ORF *slo* with removing its signal sequence; Amp^R^ | In this lab  In this lab  Invitrogen  This study  This study |
| Primers  PLY-F  PLY-R  SLO-F  SLO-R | Sequence^b^ (5′–3′)  CGGGATCCATGGCAAATAAAGCAGTAAATGAC GGGGTACCCCTAGTCATTTTCTACCTTATCCTCTACC  CCGCGGATCCGCTCCCAAAGAAATGCCACTA  CGACGAATTCCTACTTATAAGTAATCGAACCATA | PCR products  The ORF of PLY  The ORF of SLO |

**NOTE.**^a^ Amp^R^, ampicillin resistant; Cm^R^, chloramphenicol resistant; Kan^R^, kanamycin resistant ; ^b^The underlined sequences are the restriction sites
